# The Mechanical Components of the Dynein Motor

**DOI:** 10.1100/tsw.2010.76

**Published:** 2010-05-04

**Authors:** Peter Höök

**Affiliations:** Department of Pathology and Cell Biology, Columbia University, New York, NY, USA

**Keywords:** dynein, motor protein, AAA protein, stalk, microtubule

## Abstract

Unlike our understanding of the other two classes of cytoskeletal motor proteins, the myosins and kinesins, we have only recently begun to comprehend the molecular mechanism for how dynein produces force and movement. The slow progress has been attributed, in part, to the enormous size of the dynein force-producing head, but also to the complex interplay between its structural components, each of which has a unique role in regulating dynein motor activity. The integrated and highly coordinated mechanism by which these structures work together in powering the dynein machinery is discussed in this review.

## DYNEIN FUNCTION AND MOLECULAR STRUCTURE

The microtubule-associated motor protein dynein utilizes energy from ATP hydrolysis to facilitate maintenance, function, and growth in eukaryotic cells. There are two known forms of the protein: cytoplasmic dynein, which is responsible for long-range retrograde transport of cellular cargo and in a variety of mitotic functions, including spindle orientation and nucleus positioning; and axonemal dynein, for ciliary and flagellar beating. The structural layout of dynein has been remarkably conserved, despite the wide range of functional assignments associated with this motor protein. In its most abundant form, dynein assembles into a homodimeric complex with a force-producing head and a slender tail to which a variety of accessory subunits implemented in dynein stability and cargo recruitment bind (Fig. 1). The ~380-kDa head (Fig. 2) consists of six concatenated AAA ATPase subunits arranged in a ring[1,2,3] from which three structures project: the linker, which has been proposed to function as a mechanical lever during force generation[4,5]; the stalk, a fine 12- to 15-nm-long structure responsible for microtubule binding[6]; and a C-terminal region, the function of which remains poorly understood.

## MECHANISM OF DYNEIN MOVEMENT

Most of what is known about the molecular mechanism underlying dynein force production and directed movement has evolved from single-molecule studies on the cytoplasmic form of the protein. The coordinated stepping of dynein along the length of the microtubule has been characterized as hand-over-hand, i.e., while one head remains attached to the microtubule, the other head takes a predominant 16-nm

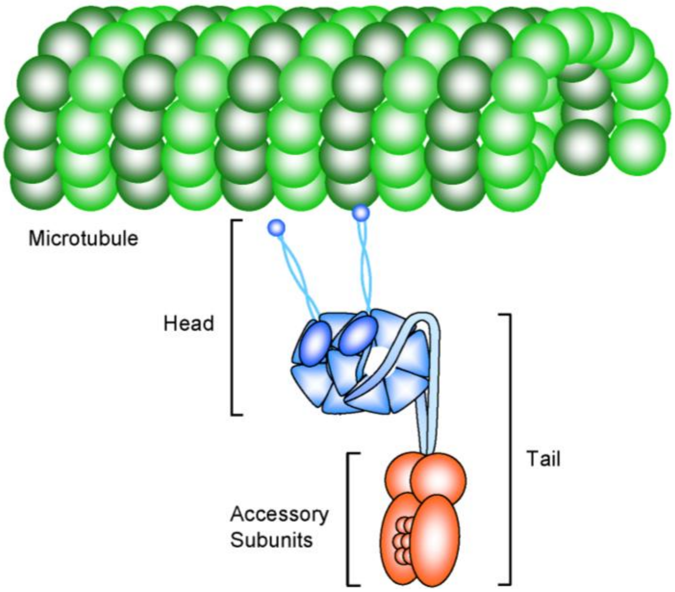

**FIGURE 1.** The cytoplasmic dynein complex. The catalytic head is responsible for microtubule binding and force production. Dynein advances along the microtubule by shuffling its heads in a hand-over-hand manner. Accessory subunits at the base of the tail domain mediate binding of intracellular cargo.

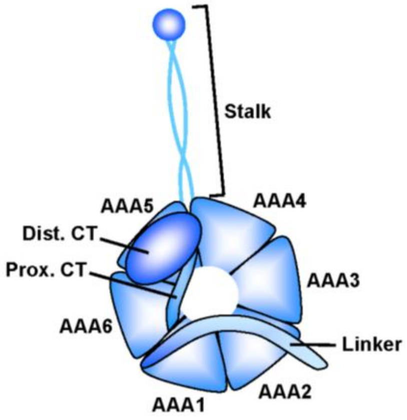

**FIGURE 2.** Depiction of the dynein head and its mechanical components based on high-resolution electron microscopy images.

step from its rear position, advances alongside the attached head, and becomes the leading head following microtubule rebinding[7,8]. The 16-nm step size results in a center-of-mass displacement of 8 nm for a dimeric dynein, although displacements of 16, 24, and even 32 nm have been reported[8,9].

Cytoplasmic dynein has the ability to take hundreds of steps along microtubules without falling off and retains its processivity under load. This ability to move for relative long distances requires the two heads to move in synchronized fashion and it remains a mystery in the field as to how this is achieved. The mechanism is thought to involve tight coupling between the mechanochemical cycles of both heads so that one head senses the state of the other, either directly through head-head interaction or by long-distance intramolecular communication (see [10] for review). Processivity and velocity of molecular motors are known to decrease with an increase in mechanical load, and single molecular analysis on bovine cytoplasmic dynein has shown that when the external force equals that produced by the dynein motor, it stalls and no further movement is observed[9]. Interestingly, stall force from yeast dynein has been reported to be four- to fivefold higher and velocity about 15-fold lower compared with its counterpart in higher eukaryotes[8,9,11,12]. A credible explanation for the difference in motor performance between lower and higher dynein has not been established. One possibility resides in the fact that a 32-kDa C-terminal extension of the dynein head is missing in the fungal form of the protein, suggesting a central role for this region in regulating some aspects of dynein motor function, which is discussed later in this review.

## THE DYNEIN MECHANOCHEMICAL CYCLE

Dynein cycles through two major mechanical states, the pre- and postpower stroke states (Fig. 3). The transition between the two states and the sequence of conformational changes leading up to the power stroke, the step at which dynein produces force and movement, have been studied in detail. Following termination of the power stroke, the dynein head remains attached to the microtubule with high affinity. Binding of ATP produces a series of structural changes that restore the head to its prepower stroke state. First, the stalk adopts a microtubule low-affinity conformation leading to rapid microtubule dissociation of the dynein head[13,14]. Immediately following microtubule dissociation, the linker shifts position on the AAA ring and assumes its prepower stroke conformation[5,15]. The shift in stalk conformation accelerates hydrolysis of ATP to ADP and Pi[16] as the head remains dissociated from the microtubule. Following release of Pi, the stalk readjusts its conformation back to its microtubule high-affinity state and the head reassociates with the microtubule surface[14,15]. The swing of the linker to its postpower stroke position subsequent to dynein rebinding is coupled with the power stroke and production of force[5,15]. Microtubule binding accelerates the pre- to poststroke transition of the linker[17] and the rate-limiting release of ADP[18,19,20]. The dissociation of ADP allows ATP to rebind and the head to enter a new mechanochemical cycle. This scheme suggests that the nucleotide state modulates the affinity by which the dynein head binds microtubules. It remains undetermined, however, how the individual AAA subunits communicate in powering changes in microtubule affinity and force generation, the subject of which is addressed below.

## THE STRUCTURAL COMPONENTS OF THE DYNEIN HEAD

In striking contrast to the kinesin family of motor proteins with its 15 structurally and functionally diverse members, cytoplasmic dynein utilizes its single structural form to carry out a wide variety of cellular functions, many of which are similar to those produced by the kinesins. Consequently, the dynein motor has been adapted to use its single machinery ingeniously. To pull off this task, dynein invokes the highly coordinated action of 11 structural elements within its catalytic head.

### AAA Ring

Dynein belongs to the AAA+ family of ATPases[1]. Members of this family have one or more copies of a highly conserved AAA domain that includes nucleotide-binding and hydrolysis-consensus sites. Unlike other AAA proteins, which typically form a ring-shaped assembly of individual monomers or dimers, the six AAA subunits in dynein are dissimilar and covalently linked. While many of the AAA consensus motifs are conserved in AAA1 to AAA4, they are partly or entirely degenerated in AAA5 and AAA6, consistent with earlier biochemical results showing binding of four ATP per dynein head[21]. This raises the question of how dynein makes use of these multiple ATP sites in controlling dynein motor activity.

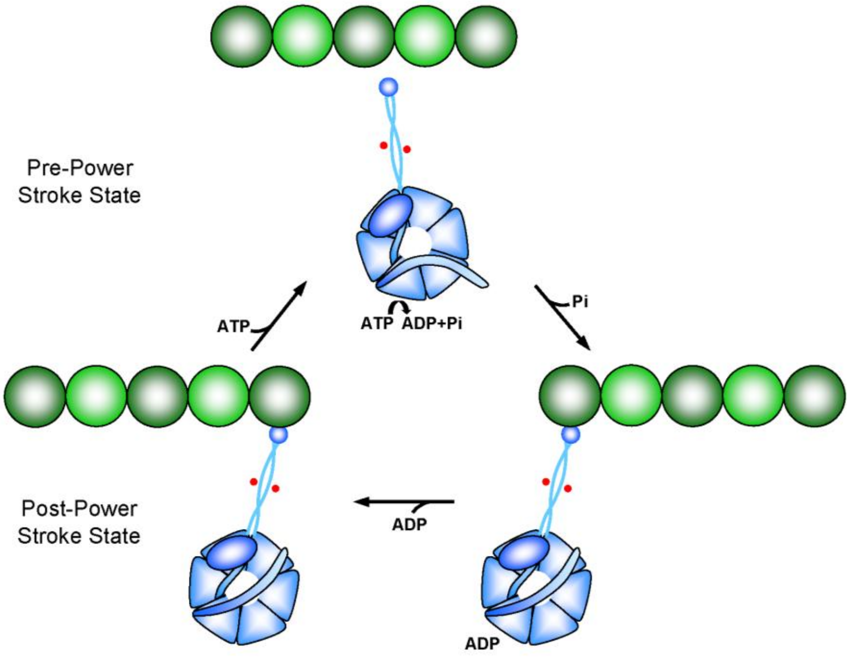

**FIGURE 3.** The transitions between the pre- and postpower stroke states are dependent on transient, cyclical, and highly coordinated interactions between the structural elements of the dynein head. Upon binding of ATP (for simplicity only ATP binding to AAA1 is shown), the stalk α-helical coiled coil undergoes a conformational shift (indicated by the red dots) that releases the dynein head from the microtubule and accelerates hydrolysis of ATP to ADP and Pi. The linker adopts its prepower stroke conformation by docking onto AAA2. Following release of Pi, the stalk resumes its high-affinity conformation that locks the dynein head onto the microtubule. The swing of the linker from its prepower stroke position at AAA2 to AAA4 triggers the power stroke and ADP is released.

The coupled interaction between AAA1 to AAA4 is complex and has been subjected to extensive attention in recent years. Studies on cytoplasmic dynein AAA mutants have highlighted the importance of multiple sites for ATP binding and hydrolysis. These studies showed that nucleotide binding at AAA1 and AAA3 are required for ATP-induced release of dynein from microtubules[22,23], and for microtubule-activated ATPase[23]. Blocking hydrolysis at AAA1 and AAA3 produced results similar to those of the nucleotide-binding mutants[24,25], although the AAA3 hydrolysis mutant had the ability to move processivily in a single molecule motility assay, albeit at significantly lower velocity compared with that of wild-type dynein[25]. Interestingly, the AAA3 domain of cytoplasmic dynein 2 – the retrograde motor found exclusively within the ciliary compartment of the cell – lacks the Walker B motif (responsible for hydrolysis) consistent with a mechanism by which nucleotide binding and hydrolysis is uncoupled in AAA3 of this dynein. Very little, if anything, is known of the mechanochemical function of dynein 2, and the potential effect of only one functional site for ATP hydrolysis remains to be tested.

Given that dynein motor activity is dependent on the nucleotide state in both AAA1 and AAA3, the eminent question arises if rate of hydrolysis and product release differ between the two AAA domains, and what implication that may have on dynein regulation. Results from steady-state ATPase kinetics implies that the dynein head releases ADP at two separate rates, each of which is stimulated by microtubules and consistent with nucleotide release from two separate ADP-binding sites[20]. Presumably, the higher rate of product release stems from AAA1, which, on the basis of vanadate photocleavage experiments, has been designated as the main hydrolysis site[6,26].

The functional significance of the second and fourth AAA subunit has not been fully established, but they seem to fine tune some aspects of dynein motor regulation. Eliminating nucleotide hydrolysis in

AAA2 and AAA4 produced a drop in basal dynein ATPase activity in one study[23], but not in another[25], and data from single-molecule experiments on yeast dynein AAA4 mutants showed only minimal effects on dynein velocity, microtubule binding, and microtubule-stimulated ATPase activity[25]. These results do not exclude the possibility that AAA2 and AAA4 provide other means of structural control than through nucleotide binding and hydrolysis. Previous sequence analysis of the AAA protein superfamily identified five conserved motifs involved in ATP binding and hydrolysis: Walker A, Walker B, sensor 1, sensor 2, and the arginine finger[1]. The positively charged arginine finger binds to the nucleotide-binding site in an adjacent AAA subunit, where it stabilizes the developing negative charge in the ATP hydrolysis transition state. The motif has been shown in other AAA proteins to be required for ATP hydrolysis[27]. Previous phylogenetic analysis of the dynein family[28] revealed that the arginine finger is missing in AAA1, as expected, but conserved in AAA2 to AAA4 in all species and forms of cytoplasmic dynein, with the exception of AAA4 in cytoplamic dynein 2. Given the limited impact of the AAA2 and AAA4 nucleotide-binding and hydrolysis motifs in regulating dynein motor function, the interfacial arginine fingers may serve in a more influential capacity by sensing and assisting nucleotide processing and to communicate this information in a cooperative, cyclical manner.

## Stalk

Three unique features characterize the dynein microtubule-binding stalk: the ability to (1) modulate its binding affinity to microtubules, (2) communicate conformational information to the catalytic sites within the AAA ring, and (3) adopt a structure capable of rapid, cyclical conformational changes. Biochemical experiments on recombinant head and stalk fragments have provided revealing new data on the mechanism by which the stalk – a ~13-nm-long antiparallel α-helical coiled coil[29] with a globular, all-helical microtubule-binding domain at its tip[6,30] – adjusts its binding affinity and communicates this information along the extended coiled coil to the remainder of the motor. Gibbons et al.[14] discovered that microtubule binding of dynein depends on a small shift in the register of the stalk's coiled coil α-helices that reorient the microtubule-binding domain into conformations of low and high microtubule binding affinity. In a subsequent analysis of the entire dynein head, the stalk was locked in low and high binding affinity conformations by disulfide cross-linking, and the motor mutants were analyzed on the basis of microtubule binding and ATPase activity[16]. Remarkably, the α-helical register of the stalk was found to correlate both with the affinity by which the head bound to microtubules and the rate by which it hydrolyzed ATP, a result that in a profound way addressed the long-standing question of how structural information from ATP binding and hydrolysis at AAA1 and AAA3 is communicated to the tip of the stalk and back in a cyclical manner.

In contrast to a typical α-helical coiled coil where the hydrophobic core forms a stabilizing entity that provides few measures of mechanical compliance, the cyclical rearrangement within the core region of the dynein stalk coiled coil suggests a partially disordered α-helical structure. This assumption finds support in the lack of consensus sequence at consecutive heptad core positions within the central segment of the coiled coil, indicating that this region may serve as a point of flexibility and conformational instability. The prevalence of canonical hydrophobic residues in the proximal and distal regions, on the other hand, is in conformity with higher propensity for stable coiled coil conformation. Physiochemical work on the coiled coil portion of the stalk provided experimental evidence for a destabilized and unstructured central region that adopts complete α-helical secondary structure in the full-length stalk[29]. This result argues for the possibility that the distal and proximal regions of the coiled coil are required for complete folding. Thus, the regions' proximity to the catalytic AAA ring and microtubule-binding domain may provide them with the ability to mediate changes in stalk flexibility and stability as dynein cycles through microtubule binding and release.

## Linker

The linker domain was previously identified in single-molecule electron microscopy images of dynein molecules as a slender structure emerging from the N-terminus of AAA1 and curled around one side of the AAA ring[4] (Fig. 2). Fluorescence resonance energy transfer experiments showed that the linker undergoes cyclical, nucleotide-dependent displacements between two sites on the AAA ring[5], which were later identified as AAA2 and AAA4[3] (Fig. 3). On the basis of kinetic data that coupled the swing of the linker with the pre- and postpower stroke state of the dynein head[15], the linker was proposed to serve in the role of a mechanical lever that amplifies small movements and assists in producing the dynein power stroke analogous to the role of similar elements in kinesin[31] and myosin[32]. Removal of the N-terminal linker sequence reduced the rate of basal ATP hydrolysis and abolished microtubule-stimulated ATPase activity[3,8], consistent with a role in accelerating nucleotide hydrolysis during the prepower stroke state, and coherent with a model by which the linker acts both as an important regulator of dynein ATPase activity and as a force-generating lever. One possibility that emerges from these results is that the docking of the linker domain onto AAA2 and AAA4 initiates conformational changes that are communicated to the nucleotide-binding and hydrolysis sites in AAA1 and AAA3 by intersubunit motifs, such as the arginine finger.

## C-Terminus

The C-terminus of dynein is maybe the most intriguing component of the motor. This is due to the fact that the C-terminus is the least investigated and unrecognized of the motor components, and the only structure that is not present in all dyneins. The C-terminus is divided into a proximal region present in all species and forms of dynein, and a larger distal region that is missing in many fungal cytoplasmic dyneins. The two regions are connected by a ~25-residue segment, which, on the basis of order/disorder structural prediction[33] and proteolytic analysis[20], appears disordered and may function as a flexible hinge. Roberts et al.[3] determined the three-dimensional position of the C-terminus by inserting blue fluorescent proteins (BFP) at the junction of the proximal and distal regions, and at the extreme end of the C-terminus in *Dictyostelium* cytoplasmic dynein head constructs. On the basis of electron microscopy images of the head mutants, the C-terminus appears to make two sharp turns, the first as it emerges from AAA6 and folds up on one side of the AAA ring, and a second near the base of the stalk, which coincides with the location of the flexible hinge. The location of the extreme C-terminal BFP at the junction of AAA5 and AAA6 positions the distal C-terminal region between the fourth and sixth AAA subunit (Fig. 2).

Although the exact role of the C-terminus is unknown, some leads into its function are provided by the lack of the distal C-terminal region in fungal cytoplasmic dynein. Functional analysis of yeast dynein has demonstrated consistently lower velocity, higher stall force, and longer run length, a measure of processivity, than mammalian dynein[8,9,11,12,34]. These results are suggestive of a model in which the distal C-terminal region accelerates the rate of dynein release from microtubules, conceivably through association with the microtubule-binding stalk. In fact, removal of the entire C-terminus in *Dictyostelium* cytoplasmic dynein generated a motor with the inability to bind microtubules[3], consistent with the proximity of this structure to the base of the stalk.

## CONCLUSION

The fundamental role for dynein in a range of cellular processes has invoked a succession of studies into the understanding of dynein mechanism and transport. Despite the challenge brought on by the magnitude and complexity of the dynein motor, these studies have provided valuable information into the various roles of the motor’s substructures. A deeper insight into the molecular mechanism by which these structures control dynein force production and movement must await structural information at atomic resolution.
